# Employee Age Alters the Effects of Justice on Emotional Exhaustion and Organizational Deviance

**DOI:** 10.3389/fpsyg.2017.00479

**Published:** 2017-04-06

**Authors:** Justin P. Brienza, D. Ramona Bobocel

**Affiliations:** Departmental of Psychology, University of WaterlooWaterloo, ON, Canada

**Keywords:** employee age, organizational justice, deviance, emotional exhaustion, instrumental and relational needs

## Abstract

Fairness in the workplace attenuates a host of negative individual and organizational outcomes. However, research on the psychology of aging challenges the assumption that fairness operates similarly across different age groups. The current research explored how older workers, vis-à-vis younger workers, react to perceptions of fairness. Integrating socioemotional selectivity theory and the multiple needs theory of organizational justice, we generated novel predictions regarding the relations between perceptions of workplace justice, emotional exhaustion, and employee deviance. Specifically, we hypothesized and found that employee age moderates the negative relation between justice facets and deviance (Study 1) and emotional exhaustion (Study 2). We also found that emotional exhaustion mediates the differential effects of justice on deviance, and that this relation depends on employee age (Study 2). Relative to younger workers, older workers are more sensitive to informational and interpersonal justice; in contrast, relative to older workers, younger workers are more sensitive to distributive and procedural justice. The research supports and extends existing theory on organizational justice and on the psychology of aging. Moreover, it highlights the importance of considering employee age as a focal variable of interest in the study of justice processes, and in organizational research more generally.

## Introduction

Fair treatment can alleviate negative psychological states, such as emotional exhaustion (e.g., Liljegren and Ekberg, 2009; Lambert et al., 2010), that deplete the self-control required to maintain job performance and inhibit counterproductive behavior (Schaufeli et al., 2009; Bolton et al., 2012). Consistent with this logic, perceptions of organizational justice show reliable negative relations with a broad family of deviant workplace behaviors (Cohen-Charash and Spector, 2001; Dalal, 2005; Jones, 2009). Nevertheless, do all experiences of justice relate similarly to these outcomes for all employees? In the present research, we suggest a novel, more nuanced understanding of how perceptions of justice relate to employee deviance and emotional exhaustion by considering the role of employee age.

The current research integrates two previously separate theoretical frameworks—the multiple needs model of justice (MNM; Cropanzano et al., 2001) and socioemotional selectivity theory of human aging (SST; Carstensen, 1995). The multiple needs model of justice suggests that fair treatment fulfills fundamental psychological needs, including the need for instrumental control and the need for relational belonging; it also suggests that different fairness-related experiences (i.e., distributive, procedural, informational, and interpersonal justice) can be differentially relevant for fulfilling such needs. As explained in the next sections, distributive and procedural justice are relatively more likely to satisfy employees' needs for instrumental control, whereas informational and interpersonal justice are more likely to satisfy needs for relational belonging (Cropanzano et al., 2001). Interestingly, socioemotional selectivity theory (Carstensen, 1995) and other research on human aging suggests that as people age, they become less concerned with instrumental needs and more motivated by relational needs.

**Figure 1:**
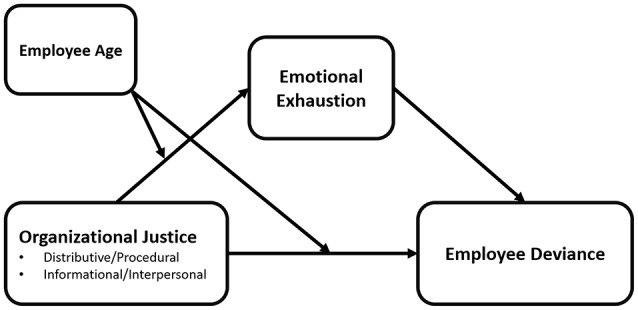
**Conceptual moderated mediation model**. Emotional exhaustion mediates the negative relations between distributive and procedural justice on deviance among younger employees; emotional exhaustion mediates the negative relations between informational and interpersonal justice on deviance among older employees.

Therefore, integrating MNM and SST, we predicted that employee age would moderate the effects of justice on employee deviance and emotional exhaustion. We focus on employee deviance and emotional exhaustion given their theoretical relationship with the fulfillment of needs. When instrumental and relational needs are satisfied, as when people experience fair treatment, negative emotional states that can increase emotional exhaustion are alleviated, leaving intact the self-regulatory resources required to maintain appropriate job behavior and suppress inappropriate job behavior. Thus, we expected that emotional exhaustion would mediate the negative relations between justice and workplace deviance, and that employee age would moderate these relations. Figure 1 depicts our theoretical model.

The current research makes several contributions. First, the findings reveal that employee age dramatically alters the relations among important organizational variables (i.e., justice, emotional exhaustion, and deviance), adding to the growing scholarship on age-related psychological changes in the organizational context. Moreover, the research underscores the importance of considering employee age in the study of organizational justice specifically; although much is known about the effects of justice on employee behavior (see Colquitt et al., 2013), little research has considered how employee age may alter justice processes. Third, the present research provides conceptual support for two theoretical frameworks—socioemotional selectivity theory and the multiple needs model of justice—and illustrates the utility of utilizing research on the psychology of aging to better understand organizational phenomena. Finally, from a practical perspective, the current research highlights the importance of “fit” between the design and enactment fairness-related policies and employee age. By demonstrating age-related dissociative relations among organizational phenomena, our research provides impetus for practitioners to examine employee age closely when enacting organizational policies intended to satisfy different employee needs.

In the next section, we review the theoretical rationale underlying the present research by drawing on the SST and MNM. Then, we derive novel hypotheses from the integration of these literatures, which we test in two studies.

## Theoretical background

### Age-related changes in needs

Socioemotional selectivity theory (SST; Carstensen, 1992) is perhaps the most prominent conceptual framework for understanding age-related shifts in human motivation, building on earlier research on value differences between younger and older people (Ryff and Baltes, 1976). SST argues that age-related changes in perspective of time and increasing relevance of emotion regulation lead to an increasing preference among people as they age for quality over quantity of social contact. Relative to younger people, older people actively select and create positive socio-emotional experiences in the service of maximizing the quality of social experience (Fredrickson and Carstensen, 1990; Lang and Carstensen, 1994; Carstensen, 1995; Carstensen and Mikels, 2005; Mather and Carstensen, 2005). As one example, in a 34-year longitudinal study, Carstensen (1992) found that social contact in relationships that serve instrumental purposes decreased with age, but social contact that fulfills quality relational needs remained stable or actually increased.

The broader psychological literature provides converging evidence for an age-related transition from instrumental to relational orientation. As people age, they increasingly prioritize positive social emotion and down-regulate negative social emotion (Gross et al., 1997), and they become more agreeable and less neurotic (Terracciano et al., 2005; Allemand et al., 2007). Similarly, with age, individuals become more empathetic (Sze et al., 2012), show improvements in reasoning about social dilemmas (Grossmann et al., 2010), and engage less in antisocial behavior (Lau et al., 2003; Tittle et al., 2003). Similar effects are observed in organizational research. For example, employee age is associated with increased motivation toward positive workplace relationships, greater cooperation and respect, increased orientation toward generative identity, and decreased motivation toward achievement, status, instrumental control, and competition in the workplace (e.g., Leviatan, 1992; Kanfer and Ackerman, 2004; Lord, 2004; Caldwell et al., 2008; Kooij et al., 2008, 2010, 2011; Stamov-Roßnagel and Biemann, 2012; Tenhiälä et al., 2013). Consistent with these latter findings, research has revealed negative relations between employee age and antisocial tendencies such as deviance, and related constructs such as revenge and retaliation (e.g., Gruys and Sackett, 2003; Lau et al., 2003; Bobocel, 2013).

In summary, evidence reveals a transition from instrumental to relational orientation as a function of age. Therefore, employee age might alter the personal relevance of workplace experiences that are associated with fulfilling instrumental and relational needs. As discussed below, despite theory suggesting that fairness can satisfy such needs, no research has integrated the literatures on aging and organizational justice to examine whether age may alter employees' sensitivity to different facets of justice.

### Justice and the fulfillment of instrumental and relational needs

Justice researchers generally distinguish between four justice concepts. Distributive justice refers to people's perceptions of the fairness of outcomes they receive, such as compensation and benefits. Procedural justice refers to people's perceptions of the fairness of the processes by which decisions are made. Informational and interpersonal justice refer to people's perceptions of the quality of treatment they receive when authorities are implementing decisions, for example, whether they received adequate explanations and respectful treatment, respectively (for review, see Colquitt et al., 2005). A large body of research on organizational justice has demonstrated that employees' perceptions of justice predict numerous organizational outcomes (Cohen-Charash and Spector, 2001; Colquitt et al., 2001, 2013; Fassina et al., 2008; Whitman et al., 2012; Shao et al., 2013; Rupp et al., 2014).

According to the MNM (Cropanzano et al., 2001; also see Lind, 2001) one reason for the pervasive impact of justice perceptions is that fairness plays a crucial role in fulfilling multiple basic human needs. For example, equity theory of distributive justice (e.g., Adams, 1965) and control theories of procedural justice (e.g., Leventhal, 1976) argued that justice can fulfill people's need for control over their own material outcomes. According to these theories, organizational justice has *instrumental value* because it maximizes one's likelihood of obtaining adequate outcomes. Later theory and research on procedural (Lind and Tyler, 1988) and interactional justice (e.g., Bies and Moag, 1986; Tyler and Bies, 1990) argued that justice can also fulfill people's needs for social belonging. According to these models, justice also has *relational value* in that it communicates that one is valued and respected by one's social network (for recent review, see Bobocel and Gosse, 2015).

### Linking justice to deviance via emotional exhaustion

From MNM and justice research, it is clear that fair treatment fulfills employees' instrumental and relational needs. From research in the broader psychological literature, it is also clear that when people perceive fulfillment of instrumental and relational needs, they experience lower levels of negative psychological states that lead to emotional exhaustion (e.g., low vitality, anxiety; Twenge et al., 2002; Ryan and Deci, 2008; Zhou et al., 2009). Emotional exhaustion, defined as a chronic state of depletion, impairs employees' ability to maintain appropriate-job related behavior and suppress inappropriate job-related behavior (Lee and Ashforth, 1996; Cropanzano et al., 2003; van Jaarsveld et al., 2010). Thus, we reasoned that experiences of fair treatment would predict lower levels of deviance via reductions in emotional exhaustion.

Organizational research supports the above reasoning. The negative association between justice and employee deviance is well-established (e.g., Aquino et al., 1999; Dalal, 2005; Berry et al., 2007; Jones, 2009; Liljegren and Ekberg, 2009; Colquitt et al., 2013; Holtz and Harold, 2013; Shao et al., 2013; Rupp et al., 2014). Meta-analyses have estimated the relations between different dimensions of justice and deviance to range from −0.22 to −0.32 (e.g., Colquitt et al., 2013). Whereas fewer studies have examined the relation between justice and emotional exhaustion, the research also indicates a negative association. For example, Lambert et al. (2010) found negative relations between distributive and procedural justice and emotional exhaustion.

Interestingly, despite early theorizing, only recently have researchers begun to examine the possible mediating role of emotional exhaustion in the relations between justice and organizational outcomes. For example, Campbell et al. (2013) demonstrated that emotional exhaustion mediates the negative relation between organizational justice and turnover (Campbell et al., 2013). Especially relevant to the current studies, Matta et al. (2014) demonstrated that state-level negative emotions mediate the negative relation between employees' perceptions of the fairness of daily events and their counterproductive workplace behavior.

In summary, there is ample reason to expect that organizational justice will relate negatively to both employee deviance and emotional exhaustion, and that emotional exhaustion will mediate the relations between justice and deviance. Nevertheless, in the present research, we also develop more fine-grained hypotheses regarding the justice-to-deviance relations by integrating MNM and SST.

### Integration and hypotheses

As explained earlier, research demonstrates that the salience of instrumental and relational needs change with age; relatedly, justice facets differentially fulfill these same needs. Employees perceive distributive justice when they believe that their outcomes are equitable; thus, distributive justice has direct instrumental value. Similarly, employees perceive procedural justice when they believe that they have control over the procedures through which outcomes are generated; thus, procedural justice also has instrumental value by affording employees indirect control over their outcomes. In contrast, employees perceived informational and interpersonal justice when they believe that authorities have adequately explained decisions and have treated them respectfully when implementing decisions; thus, relative to distributive and procedural justice, informational and interpersonal justice are more likely to satisfy employees' relational needs (for similar reasoning, see Johnson et al., 2006).

It is important to note that theory and research on the group-value model of procedural justice (Lind and Tyler, 1988; Conlon, 1993) and later the relational model of authority (Tyler and Lind, 1992) and the group engagement model (Blader and Tyler, 2003), has demonstrated that procedural justice also has relational value (for review, see Bobocel and Gosse, 2015). However, the presumed psychological function of procedural justice may depend on how it is operationalized in research. Instrumental theories of procedural justice (e.g., Leventhal, 1976, 1980) emphasized the role of *structural aspects* of decision procedures that lead to perceptions of procedural justice (e.g., consistency of procedures, accuracy of information gathered). In contrast, relational theories (e.g., Lind and Tyler, 1988; Tyler and Lind, 1992; Tyler and Blader, 2003) emphasized both the structural and *interpersonal aspects* of procedures that lead to perceptions of procedural fairness (e.g., polite and respectful treatment, justification for decision). Importantly, the most widely used measure of procedural justice in organizational research over the past 15 years (and that used in the present studies; Colquitt, 2001) operationalizes procedural justice in terms of the former, whereas the interpersonal aspects of process are subsumed within the operationalization of interactional justice. In view of this operationalization, we expected that procedural justice would be valued more for its instrumental function than for relational reasons.

Integrating MNM and SST, we suggest that, whereas younger employees should be more responsive to distributive and procedural justice, older employees should be more responsive to informational and interpersonal justice. Employee age should therefore moderate the negative relations between organizational justice and both deviance and emotional exhaustion. Therefore, we made the following predictions:

*Hypothesis 1:* Employee age will moderate the negative relations between justice perceptions and deviance, such that (a) distributive and procedural justice will negatively predict deviance for younger employees, and (b) informational and interpersonal justice will negatively predict deviance for older employees.

*Hypothesis 2:* Employee age will moderate the negative relations between justice perceptions and emotional exhaustion, such that (a) distributive and procedural justice will negatively predict emotional exhaustion for younger employees, and (b) informational and interpersonal justice will negatively predict emotional exhaustion for older employees.

Furthermore, drawing on the extant research on justice, emotional exhaustion, and deviance, we expected that employee emotional exhaustion will mediate the justice-deviance relations. Given this, and extending Hypotheses 1 and 2, we expected that the mediating role of emotional exhaustion in the justice-deviance relations will differ as a function of employee age. Therefore, we made the following moderated mediation (Baron and Kenny, 1986) hypothesis:

*Hypothesis 3*: Employee age will moderate the mediating effect of emotional exhaustion in the justice-deviance relations, such that (a) emotional exhaustion will mediate the negative relations between distributive and procedural justice perceptions and deviance for younger, but not older employees, and (b) emotional exhaustion will mediate the negative relations between informational and interpersonal justice perceptions and deviance for older, but not younger employees.

## Study 1

To begin, Study 1 investigated the moderating role of age in the justice-deviance relations (Hypotheses 1a and 1b). Note that both Studies 1 and 2 were reviewed and approved by the Human Research Ethics Committee at the University of Waterloo.

### Methods

#### Participants

One hundred and ninety-four US working adults (99 female) were recruited via Amazon.com's Mechanical Turk (MTurk) to complete an online survey for payment (Buhrmester et al., 2011; Mason and Suri, 2012; Paolacci and Chandler, 2014; Landers and Behrend, 2015). One case had incomplete data and was not included in the analyses. Participants completed the survey in reference to their current job. Average age of participants was 39.78 (*SD* = 14.20); 69% of respondents were employed full-time in a broad range of occupations (e.g., service, professional, academic); mean organization tenure was 6.96 years (*SD* = 7.60); and the median income category was $30,000–$39,000.

#### Measures

##### Justice perceptions

We assessed employees' perceptions of justice in their current workplace over the past year, using Colquitt's (2001) 20-item scale. This scale comprises four items to assess employees' perceptions of distributive justice (e.g., “Do your outcomes reflect what you have contributed to the organization?”), seven items to assess procedural justice (e.g., “Have those procedures been free of bias?”), five items to assess informational justice (e.g., “Has your supervisor communicated details in a timely manner?”), and four items to assess interpersonal justice (e.g., “Has your supervisor treated you in a polite manner?”). All items were rated on 5-point scales (1 = *To a small extent* and 5 = *To a large extent*). Cronbach's α was 0.95 for distributive justice, 0.87 for procedural justice, 0.91 for informational justice, and 0.93 for interpersonal justice.

##### Employee deviance

We assessed employee deviance using a 15-item measure, with items from Bennett and Robinson (2000) and Jones (2009). Participants reported how frequently (1 = *Never*, 4 = *Sometimes*, 7 = *Daily*) they engaged in deviant workplace behaviors over the past year. Example items include: “Put little effort into your work,” “Spent time on personal matters while at work.” Cronbach's α for this measure was 0.91.

##### Control variables: tenure, income, and gender

Employees provided demographic information including their age, and three other variables, for use as covariates in the primary regression analysis, following the recommendations of Becker (2005). First, we controlled employee tenure and income, given that these variables are likely to be correlated with employee age (e.g., Kooij et al., 2008), and may therefore serve as alternative explanations for our findings. For example, we wanted to rule out the possibility that instrumental needs become less relevant as employees age merely because such needs are already filled by greater income or organizational tenure, which are associated with age. Similarly, we controlled participant gender, given past research indicating that men are more likely than women to engage in deviance (e.g., Hollinger and Clark, 1982; Hershcovis et al., 2007), to experience higher income and longer tenure (e.g., Lefkowitz, 1994; Schneer and Reitman, 1994), and may be less attentive to violations of relational needs (Schwartz and Rubel, 2005; Carothers and Reis, 2013).

### Results

#### Preliminary bivariate correlations

As in prior research (see Colquitt et al., 2013), perceptions of informational and interpersonal justice were highly inter-correlated (*r* = 0.719, *p* = 0.001). Given that (a) these scales shared over 50% of the variance (Law et al., 1998) and (b) we had no theoretical reason to distinguish the two facets (Colquitt and Shaw, 2005; Ambrose and Schminke, 2009), we combined them into a composite to reduce multicollinearity in the analyses. Although distributive and procedural justice were also significantly inter-correlated, the subscales shared less than 50% of the variance, thus we maintained their distinction in the analyses.

At the bivariate level, distributive, procedural, and informational/interpersonal justice correlated significantly with employee deviance (see Table 1). Consistent with past research, the overall mean level of deviance was relatively low (*M* = 1.97, *SD* = 0.83); however, the distribution was not excessively skewed and was therefore left untransformed. Deviance correlated negatively with employee age, replicating past findings. As expected, gender, tenure, and income were each related to at least one of our focal variables, therefore we included them as covariates in the primary analyses.

**Table 1:** **Means (M), standard deviations (SD), and inter-correlations among study 1 variables**.

**Variable^a^**	***M (SD)***	**Age**	**Gender**	**Tenure**	**Income**	**Dist**.	**Proc**.	**Info./inter**.	**Dev**.
Age	39.78 (14.20)								
Gender^b^	1.49 (0.50)	−0.089							
Tenure^c^	6.96 (7.60)	0.524^***^	−0.063						
Income (median)	US$ 30-39k (2.63^d^)	0.272^***^	0.120	0.292^***^					
Dist.	3.55 (1.17)	0.022	0.016	0.075	0.218^**^	(0.95)			
Proc.	3.40 (0.89)	0.189^**^	−0.015	0.163^*^	0.213^**^	0.533^***^	(0.87)		
Info./inter.	3.96 (0.94)	0.144^*^	−0.172^*^	0.154^*^	0.168^*^	0.409^***^	0.581^***^	(0.94)	
Dev.	1.97 (0.83)	−0.203^**^	0.245^***^	−0.035	−0.026	−0.260^***^	−0.253^***^	−0.287^***^	(0.91)

*Dist., distributive justice; Proc., procedural justice; Info./Inter., informational/interpersonal justice composite; Dev., Deviance. Reliability estimates (α) presented in parentheses on the diagonal. Income was assessed as individual income*.

a N = 193.

b 1 = female 2 = male.

c In years.

d *$10,000 increments*.

* p < 0.05,

** p < 0.01,

*** *p < 0.001*.

#### Test of hypotheses 1a and 1b: does employee age moderate the relations between justice and deviance?

To test Hypotheses 1a and 1b, we conducted a hierarchical regression analysis with deviance as the criterion (see Table 2). Step 1 included the control variables, and explained a significant proportion of variance. Of the control variables, only employee gender predicted deviance. Step 2 included the focal mean-centered justice predictors and employee age; together these accounted for significant increment in variance explained. Distributive justice negatively predicted deviance; informational/interpersonal justice and procedural justice did not. As expected from past research, employee age negatively predicted deviance.

**Table 2:** **Unstandardized coefficients (standard error estimates in parentheses) from the hierarchical regression analysis predicting employee deviance in Study 1**.

**Predictor^a^**	**Step 1**	**Step 2**	**Step 3**
Constant	1.486^***^ (0.183)	1.372^***^ (0.176)	1.449^***^ (0.176)
Gender^b^	0.348^***^ (0.108)	0.289^**^ (0.103)	0.259^*^ (0.102)
Tenure	0.002 (0.007)	0.014 (0.008)	0.013 (0.008)
Income	−0.020 (0.021)	0.011 (0.021)	0.007 (0.020)
Dist.		−0.123^*^ (0.052)	−0.131^*^ (0.051)
Proc.		−0.090 (0.076)	−0.084 (0.077)
Info./inter.		−0.093 (0.068)	−0.123 (0.068)
Age		-0.011^*^ (0.004)	−0.010^*^ (0.004)
Dist. × age			0.010^**^ (0.003)
Proc. × age			0.001 (0.006)
Info./inter. × age			−0.011^*^ (0.005)
*R*^2^	0.054^*^	0.192^***^	0.246^**^
Δ*R*^2^		0.138^***^	0.054^**^
Δ*F*	3.556^*^	7.847^***^	4.301^**^

*All variables were mean centered. Dist., distributive justice; Proc., procedural justice; Info./Inter., informational/interpersonal justice composite*.

a N = 191.

b *1 = female 2 = male*.

* p < 0.05,

** p < 0.01,

*** *p < 0.001*.

The three focal interaction terms were entered into Step 3 of the regression analysis and accounted for significant incremental variance. There was a significant interaction between employee age and distributive justice (*B* = 0.010, *SE* = 0.003, *t* = 2.838, *p* = 0.005, 95% CI [0.003, 0.017]), however, there was no significant interaction between employee age and procedural justice (*p* = 0.833). Hypothesis 1a was therefore partially supported. In support of Hypothesis 1b, employee age interacted with informational/interpersonal justice in predicting employee deviance (*B* = −0.011, *SE* = 0.005, *t* = −2.351, *p* = 0.020, 95% CI [−0.020, −0.002]).

**Figure 2:**
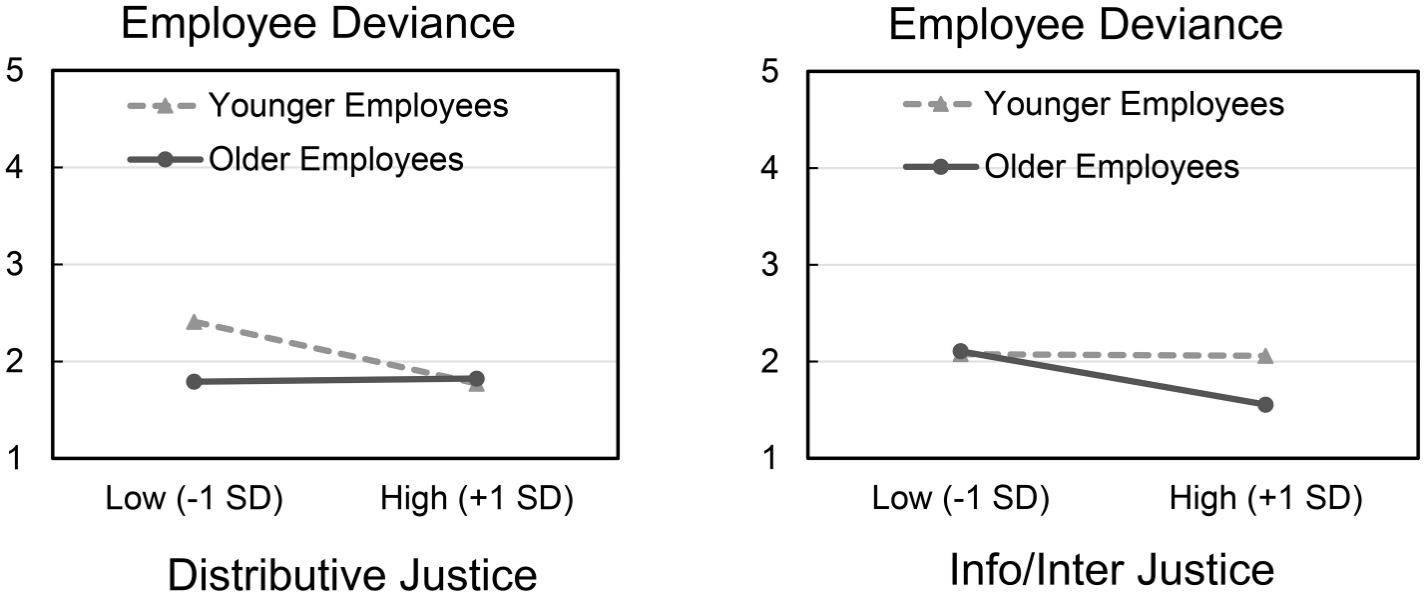
**(Left)** Study 1 interaction between age and distributive justice on employee deviance (1–5), plotted at ±1 *SD* around the means on the continuous predictors. **(Right)** Study 1 interaction between age and informational/interpersonal justice on employee deviance, plotted at ±1 *SD* around the means on the continuous predictors.

As recommended by Aiken and West (1991), we plotted the interactions at one standard deviation above and below the mean on the predictors, and simple slopes were tested for significance (Dawson and Richter, 2006). Plotting interactions at one standard deviation on employee age is appropriate, as this represents employees at approximately 26 and 54 years of age (i.e., adequately representing younger and older employees in the workplace context; US Department of Labor, 2016). As shown in Figure 2, distributive justice was significantly related to deviance in younger employees (*t* = −3.665, *p* < 0.001), but not in older employees (*t* = 0.187, *p* = 0.852). Tests of simple effects showed that the effect of employee age at −1 *SD* on distributive justice was significant (*t* = −3.869, *p* < 0.001). Also, as shown in Figure 2 informational/interpersonal justice was significantly related to deviance in older employees (*t* = −3.610, *p* < 0.001), but not in younger employees (*t* = 0.216, *p* = 0.829). Tests of simple effects showed that the effect of employee age at +1 *SD* on informational/interpersonal justice was significant (*t* = −3.107, *p* = 0.002). Importantly, the findings from Study 1 are independent of employee gender, income, and tenure^1^.

## Study 2

Study 1 provided some support for our conceptual model in which we reasoned that employee age would moderate the relations between different facets of justice and deviance. More specifically, we found partial support for Hypothesis 1a and full support for Hypothesis 1b: distributive justice predicted deviance in younger but not older employees, and informational/interpersonal justice predicted deviance in older but not younger employees. Unexpectedly, procedural justice did not interact with employee age.

The purpose of Study 2 was to replicate and extend Study 1 by examining the age-moderated mediating role of emotional exhaustion between justice perceptions and deviance (Hypotheses 2a–b and 3a–b). The fact that we predicted and observed two 2-way interactions in Study 1 renders common method variance an unlikely threat to the interpretation of the findings; nevertheless in Study 2, we utilized a two-wave survey format in which the focal variables were assessed at different times, to minimize the impact of common method variance by design (Podsakoff et al., 2003, 2012). In addition, given the consistent correlations between negative affect and employee reports of justice and deviance in the extant literature (Kaplan et al., 2009; Matta et al., 2014), consistent with other recent studies (e.g., Bobocel, 2013; Colquitt et al., 2015), we controlled for negative affect in Study 2 to increase validity. Controlling for negative affect also helps to rule out common method variance as an alternative explanation for the results (Podsakoff et al., 2003).

### Methods

#### Participants and procedure

Two hundred and thirty-one US working adults were recruited via StudyResponse.net. StudyResponse.net is an academic organization that provides researchers with access to employees who participate in online research for pay, and has been used in prior psychological research (e.g., Piccolo and Colquitt, 2006; Bobocel, 2013; also see Landers and Behrend, 2015). Study 2 was administered in two sessions, separated by approximately 2 weeks. In the first session, employees provided demographic information including the control variables (to follow) as well perceptions of organizational justice in reference to their current job. In the second session, employees completed measures of emotional exhaustion and employee deviance. Forty-one participants from the first session failed to respond to the second session. Participants who failed to respond to the second did not differ significantly from those who completed both sessions on any of the focal predictors or control variables. Two participants submitted incomplete data and were not included in the analyses. In both sessions, we included two attention check items to assess careless responding (e.g., Meade and Craig, 2012). Five participants failed both items in at least one session and were therefore excluded from analysis, leaving a total sample of 183 (85 female), for a response rate of approximately 80%. Average age was 42.15 (*SD* = 13.55). All participants were employed full-time in a broad range of occupations (e.g., service, professional, academic), as in Study 1; mean tenure was 8.96 years (*SD* = 8.04), and the median income category was $70,000–79,000.

#### Measures

##### Perceptions of organizational justice

Colquitt's (2001) measure was used, as in Study 1. Cronbach's α was 0.94 for distributive justice, 0.91 for procedural justice, 0.94 for informational justice, and 0.93 for interpersonal justice.

##### Emotional exhaustion

Emotional exhaustion was measured using 6-items from Maslach and Jackson's (1981) emotional exhaustion scale (as per Wharton and Erickson, 1995). Items assessed the degree to which employees were exhausted over the past year from their workplace experiences (e.g., “I feel used up at the end of the day,” “I feel frustrated by my job,” “I feel burned out from my work”). All items were rated on a 7-point scale (0 = *Never felt this way* to 6 = *Feel this way every day*). Following Wharton and Erickson (1995), the items were summed; scale values range from 0 to 36. Cronbach's α was 0.94.

##### Employee deviance

In Study 1, we assessed deviance broadly. Although the results supported our predictions using this broad measure, we observed higher means (and larger standard deviations) on the subset of items that referenced the organization rather than the supervisor. The majority of these items assessed *production deviance* (see Spector et al., 2006)—the failure to perform job tasks effectively. Given this, and because of the theoretical connection between emotional exhaustion and below-peak performance, in Study 2 we focused on production deviance (7 items from Study 1, plus an additional item from Gruys and Sackett, 2003). Participants reported how frequently (1 = *Never*, 4 = *Sometimes*, 7 = *Daily*) they engaged in behaviors in the past year. Example items include: “Put little effort into your work,” “Spent time on personal matters while at work,” “Spent time on non-work related tasks” “Intentionally produced lower quality work than you are capable of.” Cronbach's α for this measure was 0.94.

##### Control variables: tenure, income, gender, and negative affect

As in Study 1, employees provided demographic information including their age, tenure, income, and gender, to be used as covariates in the primary regression analysis, following the recommendations of Becker (2005). In addition, we measured negative affect for use as a control variable in the analyses (see (Podsakoff et al., 2003; Kaplan et al., 2009)). Negative affect was measured using 5 items from Watson and Clark (1999). Participants reported the extent they felt in general: angry, irritable, hostile, upset, and distressed, on a 5-point scale (1 = *Not at all* to 5 = *Extremely*). Cronbach's α for this measure was 0.91.

##### Attention-check items

We included two “instructed response items” (e.g., Meade and Craig, 2012) in both the first and second sessions. Items requested participants to select a specific response (e.g., “Please select ‘not true”’). To minimize false positives, we excluded cases from analyses only if participants failed both attention-check items in either survey).

### Results

#### Preliminary bivariate correlations

As in Study 1, informational justice and interpersonal justice items were combined given their substantial overlap, *r* = 0.781, *p* = 0.001. Distributive justice and procedural justice were also highly inter-correlated, *r* = 0.733, *p* = 0.001, and thus combined into a single index^2^.

As expected, at the bivariate level, negative affect was highly correlated with emotional exhaustion, deviance, distributive/procedural justice, and informational/interpersonal justice (Table 3), and therefore was controlled in all subsequent analyses. Deviance was significantly correlated with emotional exhaustion, informational/interpersonal justice, and marginally correlated with distributive/procedural justice. Emotional exhaustion was significantly correlated with both justice composites. As expected in light of our focus on production deviance, the mean level of deviance (*M* = 2.31) and standard deviation (*SD* = 1.24) were greater in Study 2 compared to Study 1. Employee age was again negatively associated with deviance. Given their correlations with the focal measures, we statistically controlled employee gender, tenure, and income in all subsequent analyses, as in Study 1.

**Table 3:** **Means (M), standard deviations (SD), and inter-correlations among study 2 variables**.

**Variable^a^**	***M(SD)***	**Age**	**Gender**	**Tenure**	**Income**	**Dist./proc**.	**Info./inter**.	**NA**	**EE**	**Dev**.
Age	42.15 (13.55)									
Gender^b^	1.46 (0.50)	0.021								
Tenure^c^	8.97 (8.09)	0.527^***^	0.021							
Income (median)	US$ 70-79k (2.60^d^)	−0.133	−0.183^*^	0.043						
Dist./proc.	3.49 (0.84)	−0.151^*^	−0.155^*^	0.010	0.230^**^	(0.95)				
Info./inter.	3.96 (0.90)	−0.219^**^	0.007	−0.101	0.126	0.570^***^	(0.94)			
NA	1.67 (0.77)	−0.115	−0.048	0.012	0.038	−0.213^**^	−0.271^***^	(0.91)		
EE	17.12 (7.50)	−0.113	0.054	−0.043	−0.126	−0.348^***^	−0.342^***^	0.664^***^	(0.94)	
Dev.	2.31 (1.24)	−0.192^**^	−0.128	−0.048	0.127	−0.125	−0.183^*^	0.672^***^	0.533^***^	(0.94)

*Dist./Proc., distributive/procedural justice composite; Info./Inter., informational/interpersonal justice composite; Dev., Deviance; NA, negative affect; EE, emotional exhaustion. Reliability estimates (α) presented in parentheses on the diagonal. Income was inadvertently assessed as household (vs. individual income as in Study 1)*.

a N = 183.

b 1 = female, 2 = male.

c In years.

d $10,000 increments.

* p < 0.05,

** p < 0.01,

*** *p < 0.001*.

### Tests of moderated mediation model: does employee age moderate the indirect effects (via emotional exhaustion) of justice on employee deviance?

We used Hayes's (2013) PROCESS macro for SPSS (Model 8). We used Model 8 to provide a more stringent test of our hypotheses, by testing (and controlling) for age moderation of the direct path from justice to deviance. In follow up analyses with Model 7, which does not test or control for moderation of the direct effects, the effects reported below remain statistically significant. PROCESS calculates bias-corrected bootstrapped confidence intervals (95%) at 5,000 samples for each indirect effect. PROCESS conducts regression-based path analysis and creates product terms to analyze interaction effects, centering the predictor variables prior to analysis. As PROCESS allows for a single predictor variable, we conducted two analyses, one for distributive/procedural justice, and the second for informational/interpersonal justice. We entered gender, tenure, income, and negative affect as controls (i.e., *covariates* in PROCESS; see Hayes and Preacher, 2014).

In the first test, we entered distributive/procedural justice as the predictor variable, employee age as the moderating variable, emotional exhaustion as the mediating variable, and deviance as the criterion. Consistent with Study 1, we entered informational/interpersonal justice and the age × informational/interpersonal justice product term as covariates in the model in order to control for their effect in the current analysis, thereby isolating the effect of distributive/procedural justice (see Hayes and Preacher, 2014).

**Figure 3:**
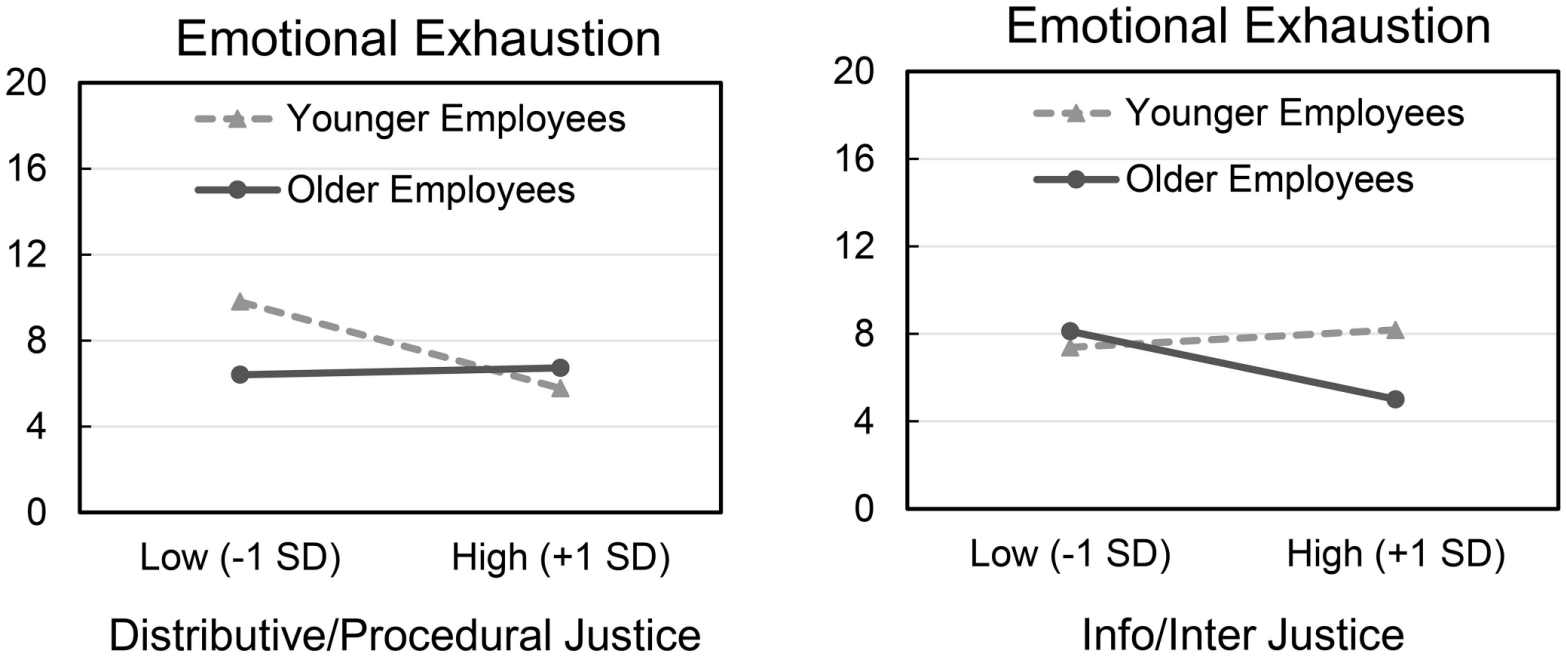
**(Left)** Study 2 interaction between age and distributive/procedural justice on emotional exhaustion (0–36), plotted at ±1 *SD* around the means on the continuous predictors. **(Right)** Study 2 interaction between age and informational/interpersonal justice on emotional exhaustion, plotted at ±1 *SD* around the means on the continuous predictors.

Results are presented in Table 4. As expected, in support of Hypothesis 2a we found that employee age interacted with distributive/procedural justice to predict emotional exhaustion (*B* = 0.094, *SE* = 0.041, *t* = 2.273, *p* = 0.024, 95% CI [0.012, 0.175]). To illustrate the interaction, we conducted a hierarchical regression (with all Study 2 control variables), and plotted the slopes as in Study 1. As shown in Figure 3, distributive/procedural justice was negatively related to emotional exhaustion in younger employees (*t* = −2.733, *p* = 0.007), but not in older employees *(p* = 0.821). Tests of simple effects showed that the effect of employee age was significant at lower (−1 *SD*) distributive/procedural justice (*t* = −2.526, *p* = 0.012), but not at higher (+1 *SD*) distributive/procedural justice (*p* = 0.478). Employee age did not moderate the direct path from distributive/procedural justice on deviance. Emotional exhaustion was a positive predictor of deviance. In support of Hypothesis 3a, we found a significant conditional indirect effect of distributive/procedural justice on deviance. Specifically, emotional exhaustion mediated the effect of distributive/procedural justice on deviance for younger but not older employees. We found no direct effects of distributive/procedural justice on employee deviance, indicating full mediation in this model. Hypothesis 3a was therefore accepted.

**Table 4:** **Unstandardized regression coefficients with confidence intervals (standard errors in parentheses) estimating emotional exhaustion and employee deviance**.

**Variable^a^**	**Emotional exhaustion (*****M*****)**		**Employee deviance (*****Y*****)**	
	**Coeff**.	**95% CI**	**Coeff**.	**95% CI**
Dist./proc. (*X*)	−**1.087 (0.593)**	−**2.258**, −**0.083**	−0.022 (0.106)	−0.187, 0.230
Emotional exhaustion (*M*)			**0.030 (0.014)**	**0.004, 0.057**
Employee age (*W*)	−0.044 (0.035)	−0.113, 0.026	−0.010 (0.006)	−0.022, 0.003
*X* × *W*	**0.094 (0.041)**	**0.012, 0.175**	−0.008 (0.007)	−0.022, 0.007
Gender	1.019 (0.790)	−0.541, 2.579	−0.215 (0.140)	−0.492, 0.062
Income	−0.206 (0.156)	−0.513, 0.101	0.049 (0.028)	−0.006, 0.103
Tenure	−0.011 (0.057)	−0.122, 0.101	0.001 (0.010)	−0.019, 0.021
Negative affect	**5.896 (0.531)**	**4.849, 6.944**	**0.864 (0.123)**	**0.621, 1.106**
Constant	7.160 (1.965)	3.281, 11.039	−0.313 (0.360)	−2.859, 2.045
	*R*^2^ = 0.545		*R*^2^ = 0.503	
	*F*_(9, 172)_ = 22.932, *p* < 0.001		*F*_(10, 171)_ = 17.306, *p* < 0.001	
	**Direct effects Coeff./95% CI**		**Indirect effects Coeff./95% CI**	
Younger employees	0.129 (0.149) [−0.166, 0.423]		−**0.071 (0.045) [**−**0.183**, −**0.007]**	
Older employees	−0.085 (0.142) [−0.366, 0.195]		−0.006 (0.027) [−0.045, 0.068]	

*Direct and indirect effects are tested at ±1 standard deviation on employee age. Significant effects are presented in bold*.

Dist./Proc., distributive/procedural justice composite. Info./Inter. and Info./Inter. × age terms are included as covariates, but are not presented here to save space.

a *N = 182*.

In the second test, we entered informational/interpersonal justice as the predictor variable, employee age as the moderating variable, emotional exhaustion as the mediating variable, and deviance as the criterion. As with the above analysis, we entered the control variables, as well as distributive/procedural justice and the age × distributive/procedural justice product term as covariates in the analysis.

Results are presented in Table 5. Consistent with the parallel analysis above, and as predicted by Hypothesis 2b, employee age interacted with informational/interpersonal justice to predict emotional exhaustion (*B* = −0.080, *SE* = 0.041, *t* = −1.982, *p* = 0.049, 95% CI [−0.160, −0.0003]). As shown in Figure 3, informational/interpersonal justice was related to emotional exhaustion in older employees (*t* = −2.667, *p* = 0.008), but not in younger employees (*p* = 0.676). Tests of simple effects showed that the effect of employee age was significant at higher (+1 *SD*) informational/interpersonal justice (*t* = −2.286, *p* = 0.023), but not at lower (−1 *SD*) informational/interpersonal justice (*p* = 0.599). Again, employee age did not moderate the direct path from informational/interpersonal justice on deviance. Emotional exhaustion was a positive predictor of deviance. In support of Hypothesis 3b, we found a significant conditional indirect effect of informational/interpersonal justice on deviance; specifically, emotional exhaustion mediated the relation between informational/interpersonal justice and deviance for older but not younger employees. We found no direct effect of informational/interpersonal justice on deviance, indicating full mediation in this model^3^. Hypothesis 3b was also accepted^4^.

**Table 5:** **Unstandardized regression coefficients with confidence intervals (standard errors in parentheses) estimating emotional exhaustion and employee deviance**.

**Variable^a^**	**Emotional exhaustion (*****M*****)**		**Employee deviance (*****Y*****)**	
	**Coeff**.	**95% CI**	**Coeff**.	**95% CI**
Info./inter. (*X*)	−0.641 (0.636)	−1.897, 0.614	−0.026 (0.113)	−0.249, 0.196
Emotional exhaustion (*M*)			**0.030 (0.014)**	**0.004, 0.057**
Employee age (*W*)	−0.046 (0.035)	−0.115, 0.024	−0.010 (0.006)	−0.022, 0.003
*X* × *W*	−**0.080 (0.041)**	−**0.160**, −**0.000**	0.002 (0.007)	−0.013, 0.016
Gender	1.019 (0.790)	−0.541, 2.579	−0.215 (0.140)	−0.492, 0.062
Income	−0.206 (0.156)	−0.513, 0.101	0.049 (0.028)	−0.006, 0.103
Tenure	−0.011 (0.057)	−0.122, 0.101	0.001 (0.010)	−0.019, 0.021
Negative affect	**5.896 (0.531)**	**4.849, 6.944**	**0.864 (0.123)**	**0.621, 1.106**
Constant	10.953 (2.898)	5.233, 16.673	0.238 (0.533)	−0.814, 1.289
	*R*^2^ = 0.545		*R*^2^ = 0.503	
	*F*_(9, 172)_ = 22.932, *p* < 0.001		*F*_(10, 171)_ = 17.306, *p* < 0.001	
	**Direct effects Coeff./95% CI**		**Indirect effects Coeff./95% CI**	
Younger employees	−0.052 (0.182) [−0.411, 0.308]		−0.014 (0.042) [−0.065, 0.100]	
Older employees	−0.001 (0.108) [−0.214, 0.212]		−**0.053 (0.028) [**−**0.123**, −**0.009]**	

*Direct and indirect effects are tested at ±1 standard deviation on employee age. Significant effects are presented in bold*.

Info./Inter., informational/interpersonal justice composite. Dist./proc. and dist./proc. × age terms are included as covariates, but not presented here to save space.

a *N = 182*.

### General discussion

The current research drew on two previously separate theoretical frameworks—socioemotional selectivity theory of human aging (Carstensen, 1995) and the multiple needs model of justice (Cropanzano et al., 2001)—to derive novel hypotheses about whether and how employee age alters the effect of perceptions of justice on employees' experiences of emotional exhaustion and deviance. Overall, the findings from the present research support our conceptual model in which we posited that employee age would moderate the relations between justice, emotional exhaustion, and deviance. Given existing evidence of age-related changes in the salience of people's needs for instrumental control and relational belonging, we predicted that age would shape employees' sensitivity to particular facets of justice that are most likely to have instrumental value and relational value. Although deviance was found to be more frequent in general among younger vs. older employees (in line with past research, e.g., Berry et al., 2007), the present research also demonstrated that employees are differentially sensitive to different forms of justice as a function of their age. Specifically, distributive and procedural justice were significant predictors of deviance and emotional exhaustion for younger (but not older) employees, whereas informational and interpersonal justice predicted deviance and emotional exhaustion for older (but not younger) employees; unexpectedly, we found no interaction between employee age and procedural justice in Study 1 (to be discussed more later). Of note, our findings are independent of participant income, organizational tenure, gender, as well as the variance explained by individual negative affect.

#### Implications for the literature on employee age and organizational sciences

In the present research, we integrated organizational justice theory with the literature on human aging. In so doing, we add to a growing body of research that demonstrates the important role of employee age for the organizational sciences in general (Baltes and Finkelstein, 2011; Bertolino et al., 2011; Tenhiälä et al., 2013; Henry et al., 2015; Scheibe et al., 2015; Zacher and Griffin, 2015), and in the study of justice processes more specifically (also see Bal et al., 2011). Our research showed that, in general, older employees engage less in employee deviance compared to their younger counterparts—but more novel, we also found that age shapes employees' sensitivity to workplace conditions, in this case, fairness-related experiences. Although fair treatment is relevant to employees of all ages, our findings suggest significant differences in the type of justice to which employees are especially sensitive. Younger employees are more sensitive than older employees to justice that fulfills needs for instrumental control, whereas older employees are more sensitive to justice that fulfills needs for relational belonging. As noted earlier, our findings are not accounted for by age-related differences in income, workplace tenure, or gender. Thus, they are consistent with research on human aging that has documented age-related changes in the salience of people's needs for instrumental control and relational belonging.

#### Implications for the literature on organizational justice and employee deviance

The present research has several important implications for research on organizational justice and employee deviance. Although past research has demonstrated a reliable negative relation between organizational justice and deviance (Cohen-Charash and Spector, 2001; Colquitt et al., 2001, 2013; Dalal, 2005; Berry et al., 2007), we advance the literature by demonstrating that different facets of justice relate to production deviance for younger and older employees. Similarly, whereas past research has long argued for the mediating role of emotional exhaustion in the association between justice and deviance, only a few recent studies have examined this relation empirically (e.g., Matta et al., 2014). In Study 2, we found that the negative relation between justice and production deviance can be explained through the effect of justice on emotional exhaustion. Moreover, employee age moderated this mediated effect: Older employees were more emotionally exhausted and in turn less effective performers when they perceived lower informational-interpersonal justice; in contrast, younger employees were more emotionally exhausted and in turn less effective when they perceived lower distributive/procedural justice. A recent meta-analysis revealed a broad range in effect sizes for different facets of justice and employee deviance (Colquitt et al., 2013), which could indicate the presence of significant uninvestigated moderators. The current research suggests that employee age may be one such moderator and reveals the existence of a more complex set of relations between justice, emotional exhaustion, and production deviance than previously known, relations that may be obscured without considering employee age. Thus, these studies contribute to a growing body of work showing the importance of investigating the effect of employee age in organizations, in particular showing the importance of considering age differences in the organizational justice research. In fact, whereas most justice research has treated employee age as a control variable, in the current research we demonstrate that employee age can dramatically alter the relations between different experiences of justice and important organizational outcomes.

Our findings also have broader implications for justice theory. In particular, they provide indirect support for the multiple needs model of justice (Cropanzano et al., 2001), which suggests that justice fulfills needs for instrumental control and relational belonging (also see Lind, 2001), and that different fairness-related experiences may be especially relevant for satisfying these needs. In this way, the present research may provide a conceptual framework for future research examining differential effects of justice facets. To the extent that distributive and procedural justice have greater instrumental value relative to informational and interpersonal justice, whereas the latter have greater relational value, then other factors (e.g., certain leadership styles) that increase the salience of employees' instrumental vs. relational needs should moderate the impact of the justice facets, as we observed in the present research.

#### Strengths and limitations

A key strength of the present research is the general convergence in findings across the two studies. Study 2 was designed as a constructive replication of Study 1, which allowed us to determine whether similar results would be observed within a different sample of employees and with a different survey design (time lagged vs. cross-sectional). As well, Study 2 extended Study 1 by examining mediation of the moderating effect of age. Although procedural justice did not have the expected role in Study 1, the results were similar, and in line with our conceptual model. Moreover, in Study 2, age did not moderate the direct path from justice to deviance when emotional exhaustion was included; nevertheless, our higher-order moderated mediation model was supported. Future research should investigate the conditions under which age determines the direct relations between justice and organizational outcomes.

Our research also has some key limitations. First, the data are correlational, and therefore causal inference is not permitted. Nevertheless, the results are consistent with our conceptual model, which we derived by integrating theory and prior research in different domains, justice, emotional exhaustion, deviance, and the psychology of aging. Moreover, in Study 2, we took steps to reduce third-variable alternative explanations, including using a two-wave study design and controlling for negative affect. Still, future research is needed to replicate our findings using experimental or longitudinal research designs, which enable causal inference among the variables.

In addition, employees may have exaggerated reports of unfairness and underreported the frequency of deviance given that our measures are self-reported. However, preliminary examination of the data revealed that responses to all measures in both studies were normally distributed and not excessively skewed; since responses were anonymous, threat of inflated or deflated reports is reduced (see Berry et al., 2012). Furthermore, our methods followed precedent in the measurement of justice, emotional exhaustion, and deviance. In particular, researchers have suggested that self-reports of deviance can be more reliable than other-reports because deviant behaviors are more likely to be acted out in private (e.g., Fox et al., 2007; Jones, 2009). Indeed, a recent meta-analysis comparing other- vs. self-reported deviance showed moderate-to-strong relations between the two, and recommended that self-reports are appropriate in most cases (Berry et al., 2012).

#### Unanswered questions for future research

Our research also raises questions for future research. First, we hypothesized that employee age would moderate the relations between justice perceptions and psychological and behavioral outcomes due to age-related changes in the relative salience of basic psychological needs. Although we derived our hypotheses from existing theory and research, we did not assess the relative salience of instrumental and relational needs in the present research. Given this, we cannot make firm conclusions regarding the underlying role of psychological needs from the present research. Whereas the pattern of our data, the double dissociations in particular, are consistent with the underlying theory, it is necessary in future research to investigate directly the extent to which the salience of needs (e.g., across time or contexts) shapes employees' sensitivity to fairness.

Second, the theory guiding our research suggested that distributive and procedural justice should have similar effects, given their instrumental value to employees, as should informational and interpersonal justice, given their relational value. Thus, we had no reason *a priori* to separate the former, nor the latter justice facets. Nevertheless, in Study 1, distributive and procedural justice were only moderately inter-correlated, therefore, on empirical grounds we analyzed them separately, demonstrating the predicted effects for distributive but not procedural justice. In a follow up analysis, we created a distributive/procedural justice composite and re-ran the regression predicting deviance. Here, we found a significant and nearly identical interaction (*t* = 2.819, *p* = 0.005) with employee age, as reported in Study 2. Thus, although distributive and procedural justice were not as highly inter-correlated in Study 1 compared to Study 2, the findings are the same as in Study 2 when we combine them in the analysis. This supplementary analysis is supportive of Hypothesis 1a, but the inconsistency in results across the two studies—in particular, the differences in magnitude of inter-correlation between distributive and procedural justice—is a limitation (but see Footnote 2). Future research is needed to better understand under what circumstances, and to what extent, employee age alters the effect of different justice facets.

Third, whereas Study 1 used a broad measure of deviance, in Study 2 we focused on production deviance specifically. We made this adjustment in Study 2 because production deviance is more frequent than the more anti-social types of deviance (e.g., abuse, theft), especially among older employees. Furthermore, whereas emotional exhaustion should be sufficient to impair employees' ability to maintain appropriate job-related behavior, we expected that the more anti-social types of deviance may require additional motivational mechanisms, such as the desire for revenge (see Footnote 4). Thus, we reasoned that production deviance would be especially pertinent to our model. Although this adjustment provided greater specificity in Study 2, the inconsistency between the two studies remains. Future research is needed to examine whether employee age also alters the effect of justice on motivational mechanisms such as the desire for revenge, which tend to amplify the more anti-social types of deviance.

Fourth, we measured employees' perceptions of justice using an established measure, which assesses the extent to which respondents perceive that particular normative rules are upheld. Recent research suggests the utility in also assessing perceived *injustice*, that is, the extent to which respondents perceive that normative fairness rules are violated (e.g., Colquitt et al., 2015). Future research is needed to explore whether and the extent to which employee age also moderates the effects of perceived injustice. For example, it may be the case that older workers, although better able to suppress deviance in general, are more sensitive than younger workers to injustice, potentially because people become more motivated to pursue prosocial experiences as they age. In this case, the differences in reactions between younger and older employees to *injustice* may be even more pronounced than those we observed here.

Considering injustice may also have implications for studying the more anti-social types of deviance, discussed earlier. That is, given that people experience losses more intensely than gains (e.g., Kahneman and Tversky, 1979) justice violations should be experienced more intensely than justice adherence. As such, justice violations would be more likely to motivate the anti-social types of deviance. Indeed, Colquitt et al. (2015) found initial evidence that injustice explained incremental variance beyond justice on one type of anti-social deviance (i.e., supervisor-directed deviance). Thus, it is possible that our present conceptual model would also apply to the more anti-social types of deviance, when assessing injustice as the predictor. Future research should continue to test the differential effects of justice and injustice experiences on the varieties of employee deviance.

Finally, future research should examine other personal or situational factors that affect the salience of employees' instrumental relative to relational needs that may moderate the impact of the different facets of justice, similar to our findings with employee age. For example, personality characteristics (e.g., strong other-orientation), may relate to greater needs for relational belonging, and therefore to greater sensitivity to informational and interpersonal justice, relative to distributive and procedural justice. Importantly, much research in psychology has demonstrated that individuals' self-identities can shift as a function of situational factors (e.g., Markus and Kitayama, 1991; Turner et al., 1994). For example, employees' collective (other-oriented) self-identity is heightened in the presence of charismatic vs. transactional leadership (see Paul et al., 2001; Zdaniuk and Bobocel, 2015). Thus, it is possible that justice may have differential effects *within*-person, depending on the context. Future research is needed to examine differential justice effects both between and within-person.

#### Practical implications

Our findings have practical importance for organizational policy and decision-making. In particular, whereas managers need to understand the importance of distributing outcomes fairly and using fair decision-making procedures, our results suggest that they also need to recognize the increasing importance of ensuring informational and interpersonal justice, within the context of an aging workforce. The current findings also highlight that, despite the importance of all forms of fair treatment, it is important to consider the “fit” between fairness-related policies and employee age. Specifically, equitable pay and fair procedures may be insufficient to satisfy older employees, in the context of an organizational climate that fails to emphasize respectful treatment. Similarly, a respectful climate may be insufficient to satisfy younger workers, in the context of relatively less equitable pay.

Beyond implications for fairness-related policies, the findings could have implications for the success of any policies that fulfill employees' instrumental or relational needs, given that reactions to such policies might differ as a function of employee age. In particular, by considering age-related changes in the salience of needs, practitioners may gain insight into why such policies are (or are not) effective. It may also suggest ways to make certain that policies appeal to a broader range of employees. For example, past research showed that older employees are less motivated than younger employees to participate in training programs (Kooij et al., 2011); drawing on the present findings, it is possible that this may be because organizations mainly promote such programs by referencing their instrumental value for career advancement, which may appeal less to older employees. Rather, organizations might increase the appeal of training programs among older employees by highlighting the relational value of training. For example, practitioners could emphasize that training can provide opportunities for older employees to mentor junior colleagues, which may be appealing in light of their older employees' orientation toward generativity. Thus, by considering employee age and age-related changes in the salience of instrumental vs. relational needs, practitioners may be better able to manage a number of organizational policies.

## Conclusion

By integrating socioemotional selectivity theory, research of human aging, and the multiple needs theory of justice, we developed and tested a novel, more nuanced understanding of the relations among organizational justice, emotional exhaustion, and employee deviance. Whereas fairness is relevant to employees of all ages, our findings suggest that there are significant differences in the type of justice to which employees are especially sensitive, as a function of age. Overall, our research has scientific and practical value, and contributes to a growing literature aimed at better understanding and improving important workplace phenomena by considering employee age.

## Ethics statement

The present research was conducted as part of JB's master's degree. This research was carried out in accordance with the recommendations of the Human Research Ethics Committee at the University of Waterloo, with written informed consent in accordance with the Declaration of Helsinki.

## Author contributions

JB provided the initial study concept. JB and DB contributed to the design. JB collected the data and conducted data analysis in collaboration with DB. JB drafted the manuscript. DB and JB revised manuscript and approved final copy for submission.

## Funding

This work was supported by the Social Sciences and Humanities Research Council of Canada Insight Grant 435-2012-0306 awarded to DB.

### Conflict of interest statement

The authors declare that the research was conducted in the absence of any commercial or financial relationships that could be construed as a potential conflict of interest.

## Footnotes

^1^Two extreme cases on deviance were observed (studentized residuals of 4.229 and 3.975) and excluded from the present analyses as outliers (Cohen et al., 2013, pp. 410–415). In follow up regressions with the outliers included, the distributive justice × age interaction remained statistically significant; the informational/interpersonal justice × age interaction was weaker (*p* = 0.102), albeit the same pattern. The differences may suggest that variables other than justice and employee age impact extreme levels of deviance.

^2^Although distributive and procedural justice were more highly inter-correlated in Study 2 than in Study 1, the magnitudes of association in the present research are within the range reported in recent meta-analytic reviews: *r*_*c*_ = 0.61; 95% CI: 0.35, 0.88 (Colquitt et al., 2013; also see Hauenstein et al., 2001).

^3^One extreme case on emotional exhaustion was observed (studentized residual of 3.406) and excluded from the present analyses as an outlier (Cohen et al., 2013, pp. 410–415). In follow up PROCESS moderated mediation tests with the outlier included, the distributive justice × age interaction was slightly weaker (*p* = 028); the informational/interpersonal justice × age interaction was slightly stronger (*p* = 0.047).

^4^Spector et al. (2006) argued that deviance falls into several “types” that are differentially, and even multiply, determined. Whereas we predicted that production deviance should be associated with emotional exhaustion, other types of deviance (e.g., abuse, theft) are likely to be determined by additional factors such as the desire for revenge (e.g., Jones, 2009). Moreover, drawing on socioemotional selectivity theory, older employees should be especially unlikely to engage in anti-social types of deviance. Nevertheless, in Study 2, we also assessed some of these types of deviance (e.g., peer-abuse, supervisor-abuse, theft) to explore whether our model would hold. Interestingly, follow up tests of moderated mediation revealed that indeed emotional exhaustion was not a significant mediator for these other types of deviance. Supplementary analyses are available on request from the first author.
